# Maternal Hepatitis B Virus Infection and Pregnancy Outcomes of Freeze-Thaw Embryo Transfer

**DOI:** 10.1001/jamanetworkopen.2023.23495

**Published:** 2023-07-14

**Authors:** Ling-Ling Ruan, Ming-Xing Chen, Enoch Appiah Adu-Gyamfi, Li-Hong Geng, Li-Juan Fu, Qi Wan, Yu-Bin Ding

**Affiliations:** 1 Department of Obstetrics and Gynecology, Women and Children’s Hospital of Chongqing Medical University, Chongqing, China; 2 Joint International Research Laboratory of Reproduction and Development of the Ministry of Education of China, School of Public Health, Chongqing Medical University, Chongqing, China; 3 Department of Biomedical Sciences, State University of New York at Albany; 4 The Reproductive Center, Chengdu Xinan Gynecology Hospital, Chengdu, China; 5 Academician Workstation, Department of Pharmacology, Changsha Medical University, Changsha, China; 6 Department of Obstetrics and Gynecology, West China Second Hospital, Sichuan University (Key Laboratory of Birth Defects and Related Diseases of Women and Children, Ministry of Education), Chengdu, China

## Abstract

This cohort study assesses the association of maternal hepatitis B virus (HBV) serostatus with pregnancy outcomes in women undergoing freeze-thaw embryo transfer (FET).

## Introduction

Hepatitis B virus (HBV) infection poses a significant global public health challenge. In mainland China, the prevalence of hepatitis B surface antigen (HBsAg) among pregnant women was estimated at 6.27% from 2013 to 2020.^1^

Infection with HBV has been linked to a higher risk of miscarriage and preterm birth in natural pregnancies.^2^ However, its effects on assisted reproductive technology, especially freeze-thaw embryo transfer (FET), are uncertain.^3^ Previous research has not explored the association between HBV infection and pregnancy outcomes in FET. To fill this knowledge gap, we conducted a retrospective cohort study to examine the association of maternal HBV serostatus with pregnancy outcomes in women undergoing FET.

## Methods

This retrospective cohort study was approved by the Ethics Committees of Chongqing Medical University and Chengdu Xinan Gynecology Hospital. A waiver of informed consent was granted because this study used deidentified data. The study followed the STROBE reporting guideline.

We investigated the association between HBV infection and pregnancy outcomes among women undergoing their first FET between January 1, 2018, and April 30, 2021, using data from Chengdu Xinan Gynecology Hospital and Chengdu Jinjiang Hospital for Women's and Children’s Health. The study focused on 3 groups: A (HBsAg positive and hepatitis Be antigen [HBeAg] negative), B (HBsAg positive and HBeAg positive), and C (HBsAg negative and HBeAg negative). Propensity score matching was used to address baseline differences among groups. The primary outcome was live birth, and secondary outcomes included biochemical pregnancy, clinical pregnancy, ectopic pregnancy, miscarriage (early and late), and preterm delivery of singleton (gestational age <37 weeks). Multivariate logistic regression was used to calculate each outcome’s adjusted odds ratios and 95% CIs. The eMethods in Supplement 1 contains the various covariates used in the regression analysis. Statistical analyses used SPSS, version 25.0 (IBM Corp), with 2-sided *P* < .05 indicating statistical significance.

## Results

After propensity score matching, 642 women in group A vs 2565 in group C, 151 in group B vs 600 in group C, and 480 in group A vs 146 in group B were included in the analysis, with partial duplication among groups. The groups did not differ regarding age, body mass index, infertility type, or cause of infertility (Table 1). In the analyses of 3 matched groups (A vs C, B vs C, and A vs B) presented in Table 2, no statistically significant differences were observed for the primary pregnancy outcome of live birth (groups A vs C odds ratio [OR], 1.13 [95% CI, 0.95-1.34]; groups B vs C OR, 1.12 [95% CI, 0.78-1.60]; groups A vs B OR, 1.25 [95% CI, 0.86-1.82]) or the secondary outcomes. These findings remained nonsignificant even after adjusting for various factors.

**Table 1.** Comparison of Patient Characteristics and Reproduction-Related Clinical Parameters Among All Groups^a^

Characteristic	Group A vs group C			Group B vs group C			Group A vs group B		
	Group A (n = 642)	Group C (n = 2565)	*P* value	Group B (n = 151)	Group C (n = 600)	*P* value	Group A (n = 480)	Group B (n = 146)	*P* value
Age, mean (SD), y	31.53 (3.54)	31.53 (3.61)	.38	29.69 (3.60)	29.62 (3.57)	.79	30.55 (3.34)	29.84 (3.52)	.03
BMI, mean (SD)	21.81 (2.96)	21.79 (2.95)	.70	21.81 (2.75)	21.81 (3.07)	.17	21.74 (3.00)	21.84 (2.77)	.70
Infertility type, No. (%)									
Primary infertility	292 (45.48)	1161 (45.26)	.92	81 (53.64)	329 (54.83)	.79	243 (50.63)	76 (52.05)	.76
Secondary infertility	350 (54.52)	1404 (54.74)		70 (46.36)	271 (45.17)		237 (49.38)	70 (47.95)	
Cause of infertility, No. (%)									
Tubal factor	474 (73.83)	1932 (75.32)	.44	114 (75.50)	451 (75.17)	.93	366 (76.25)	109 (74.66)	.69
Ovulatory disorders	10 (1.56)	33 (1.29)	.60	3 (1.99)	13 (1.99)	.89	3 (0.63)	8 (5.48)	.76
PCOS	57 (8.88)	203 (7.91)	.42	34 (22.52)	133 (22.17)	.93	57 (11.88)	29 (19.86)	.01
EMs	25 (3.89)	99 (3.86)	.97	4 (2.65)	15 (2.50)	.92	13 (2.71)	4 (2.74)	.98
Male factor	123 (19.16)	442 (17.23)	.25	25 (16.56)	110 (18.33)	.61	85 (17.71)	25 (17.12)	.87
Unexplained	15 (2.34)	60 (2.34)	.10	1 (0.66)	5 (0.83)	.83	5 (1.04)	1 (0.68)	.70
Duration of infertility, median (IQR), y	3 (2-5)	3 (2-5)	.62	3 (2-5)	3 (2-5)	.32	3 (2-5)	3 (2-5)	.88
AMH level, median (IQR), ng/mL	3.49 (1.81-5.74)	3.52 (1.89-5.83)	.65	4.94 (2.74-7.69)	5.13 (2.91-7.97)	.44	3.96 (2.13-6.25)	4.69 (2.68-7.44)	.01
FSH level, median (IQR), mIU/mL	7.45 (6.35-8.83)	7.48 (6.39-8.91)	.93	7.20 (6.16-8.50)	7.15 (6.03-8.31)	.72	7.39 (6.29-8.68)	7.21 (6.17-8.64)	.23
LH level, median (IQR), mIU/mL	4.57 (3.31-6.23)	4.14 (3.08-5.82)	.001	4.43 (3.51-6.72)	4.57 (3.31-6.70)	.69	4.51 (3.36-6.25)	4.37 (3.44-6.30)	.52
Estradiol level, median (IQR), pmol/L	49 (37-66)	48 (36-63)	.06	47 (37-63)	50 (37-63)	.54	49 (37-65)	46 (36-62)	.32
Progesterone level, median (IQR), ng/mL	0.61 (0.41-0.90)	0.58 (0.39-0.86)	.15	0.66 (0.45-0.96)	0.60 (0.43-0.88)	.23	0.62 (0.43-0.92)	0.67 (0.45-1)	.39
Endometrial thickness on the day of planning FET, median (IQR), mm	9.00 (8.50-10.50)	9.00 (8.50-10.50)	.75	9.50 (8.50-10.50)	9.50 (8.50-10.25)	.87	9.00 (8.50-10.50)	9.50 (8.50-10.50)	.65
FET protocol, No. (%)									
Natural cycle	43 (6.70)	190 (7.41)	.92	8 (5.30)	32 (5.33)	.96	30 (6.25)	8 (5.48)	.85
DRC	59 (9.19)	239 (9.32)		10 (6.62)	33 (5.50)		41 (8.54)	10 (6.85)	
HRC	481 (74.92)	1892 (73.76)		113 (74.83)	458 (76.33)		355 (73.96)	109 (74.66)	
OPC	59 (9.19)	244 (9.51)		20 (13.25)	77 (12.83)		54 (11.25)	19 (13.01)	
No. of embryos transferred, No. (%)									
1	204 (31.78)	842 (32.83)	.61	39 (25.83)	181 (30.17)	.30	144 (30.00)	38 (26.03)	.36
2	438 (68.22)	1723 (67.17)		112 (74.17)	419 (69.83)		336 (70.00)	108 (73.97)	
Type of embryo transferred, No. (%)									
Blastocyst	507 (78.97)	2043 (79.65)	.70	124 (82.12)	497 (82.83)	.84	392 (81.67)	120 (82.19)	.89
Cleavage	135 (21.03)	522 (20.35)		27 (17.88)	103 (17.17)		88 (18.33)	26 (17.81)	
No. of good quality embryos transferred, No. (%)									
0	171 (26.64)	668 (26.04)	.94	36 (23.84)	146 (24.33)	.89	119 (24.79)	35 (23.97)	.97
1	215 (33.49)	857 (33.41)		54 (35.76)	202 (33.67)		169 (35.21)	51 (34.93)	
2	256 (39.88)	1040 (40.55)		61 (40.40)	252 (42.00)		192 (40.00)	60 (41.10)	

Abbreviations: AMH, antimüllerian hormone; BMI, body mass index (calculated as weight in kilograms divided by height in meters squared); DRC, downregulation cycle; EMs, endometriosis; FET, freeze-thaw embryo transfer; FSH, follicle-stimulating hormone; HRC, hormone replacement cycle; LH, luteinizing horomone; OPC, ovulation-promoting cycle; PCOS, polycystic ovary syndrome.

SI conversion factor: To convert to nmol/L, multiply by 3.18.

^a^ Group A indicates hepatitis B surface antigen (HBsAg) positive and hepatitis Be antigen (HBeAg) negative; group B, HBsAg positive and HBeAg positive; and group C, HBsAg negative and HBeAg negative. The variables in the propensity score–matched model include age of women, body mass index, duration of infertility, type of infertility, basal sex hormone level (estradiol, progesterone, FSH, and LH), AMH level, endometrial preparation protocols, endometrial thickness, the number of good-quality embryos transferred, and the type of embryos transferred.

**Table 2.** Pregnancy Outcomes Among Groups^a^

Outcome, No. (%)	Comparison group	Reference group	*P* value	Unadjusted analysis		Adjusted analysis	
				OR (95% CI)	*P* value	OR (95% CI)	*P* value
Groups A (n = 642) vs C (n = 2565 [reference])^b^							
Live birth	323 (50.31)	1212 (47.25)	.17	1.13 (0.95-1.34)	.17	1.13 (0.95-1.34)	.19
Biochemical pregnancy	447 (69.63)	1708 (66.59)	.14	1.15 (0.95-1.39)	.14	1.14 (0.94-1.38)	.17
Clinical pregnancy	395 (61.53)	1483 (57.82)	.09	1.17 (0.98-1.39)	.09	1.16 (0.97-1.39)	.10
Ectopic pregnancy	5 (0.78)	22 (0.86)	.75	0.85 (0.32-2.26)	.75	0.85 (0.32-2.25)	.74
Early miscarriage	59 (9.19)	224 (8.73)	.93	0.99 (0.72-1.35)	.93	1.00 (0.73-1.36)	.10
Late miscarriage	8 (1.25)	27 (1.05)	.79	1.12 (0.50-2.47)	.79	1.07 (0.48-2.38)	.87
Singleton preterm delivery	44 (6.85)	133 (5.19)	.12	1.35 (0.92-1.96)	.12	1.38 (0.94-2.01)	.10
Groups B (n = 151) vs C (n = 600 [reference])^c^							
Live birth	85 (56.29)	321 (53.50)	.54	1.12 (0.78-1.60)	.54	1.12 (0.78-1.60)	.54
Biochemical pregnancy	117 (77.48)	432 (72.00)	.17	1.34 (0.88-2.04)	.18	1.33 (0.88-2.04)	.18
Clinical pregnancy	99 (65.56)	383 (63.83)	.69	1.08 (0.74-1.57)	.69	1.08 (0.74-1.57)	.70
Ectopic pregnancy	1 (0.66)	5 (0.83)	.81	0.77 (0.09-6.68)	.81	0.73 (0.08-6.33)	.77
Early miscarriage	12 (7.95)	47 (7.83)	.97	0.99 (0.50-1.94)	.97	0.99 (0.50-1.94)	.97
Late miscarriage	1 (0.66)	11 (1.83)	.29	0.35 (0.04-2.71)	.31	0.35 (0.04-2.73)	.32
Singleton preterm delivery	11 (7.28)	28 (4.67)	.11	1.88 (0.87-4.07)	.11	2.04 (0.93-4.49)	.08
Groups B (n = 146) vs A (n = 480 [reference])^d^							
Live birth	85 (58.22)	253 (52.71)	.24	1.25 (0.86-1.82)	.24	1.20 (0.82-1.76)	.34
Biochemical pregnancy	114 (78.08)	342 (71.25)	.10	1.44 (0.93-2.23)	.11	1.35 (0.86-2.12)	.19
Clinical pregnancy	97 (66.44)	304 (63.33)	.49	1.15 (0.78-1.69)	.50	1.06 (0.71-1.58)	.78
Ectopic pregnancy	1 (0.68)	5 (1.04)	.67	0.62 (0.07-5.40)	.67	0.46 (0.05-4.21)	.49
Early miscarriage	10 (6.85)	40 (8.33)	.46	0.76 (0.36-1.58)	.46	0.74 (0.35-1.56)	.43
Late miscarriage	1 (0.68)	6 (1.25)	.54	0.52 (0.06-4.35)	.54	0.48 (0.06-4.14)	.50
Singleton preterm delivery	11 (7.53)	34 (7.08)	.83	1.09 (0.51-2.34)	.83	1.10 (0.51-2.37)	.81

^a^ Group A indicates hepatitis B surface antigen (HBsAg) positive and hepatitis Be antigen (HBeAg) negative; group B, HBsAg positive and HBeAg positive; and group C, HBsAg negative and HBeAg negative.

^b^ The multiple logistic regression model was adjusted for propensity scores (age of women, body mass index, duration of infertility, type of infertility, basal sex hormone level [estradiol, progesterone, follicle-stimulating hormone (FSH), and luteinizing horomone (LH)], antimüllerian hormone [AMH] level, endometrial preparation protocols, endometrial thickness, the number of good-quality embryos transferred, and the type of embryos transferred) and luteinizing hormone level.

^c^ The multiple logistic regression model was adjusted for propensity scores (age of women, body mass index, duration of infertility, type of infertility, basal sex hormone level [estradiol, progesterone, FSH, and LH], AMH level, endometrial preparation protocols, endometrial thickness, the number of good-quality embryos transferred, and the type of embryos transferred).

^d^ The multiple logistic regression model was adjusted for propensity scores (age of women, body mass index, duration of infertility, type of infertility, basal sex hormone level [estradiol, progesterone, FSH, and LH], AMH level, endometrial preparation protocols, endometrial thickness, the number of good-quality embryos transferred, and the type of embryos transferred), age of women, and AMD level.

## Discussion

Previous studies have found no link between maternal HBV infection and live birth, preterm birth, or pregnancy rates in women undergoing assisted reproductive technology.^4,5^ Our study confirms that maternal HBV serostatus is not associated with pregnancy outcomes in infertile women undergoing FET. However, HBV infection was significantly correlated with miscarriage and preterm birth during natural pregnancy.^2^ This correlation may be due to the accumulation of HBV DNA in the placenta and trophoblast cells that may lead to placental inflammation.^2^ Pregnant women with a high HBV viral load are at risk of transmitting the virus to the developing embryo, causing adverse pregnancy outcomes.^6^ Study limitations include the retrospective design, potentially introducing bias. Additionally, only the first FET cycle was analyzed, limiting the ability to assess the long-term effects of maternal HBV infection on pregnancy outcomes. In conclusion, maternal HBV serostatus was not associated with FET pregnancy outcomes. These findings suggest that women with HBV infection can safely undergo FET, which may reduce the psychological burden of infertile couples with HBV.

## Notes

Supplement 1. **eMethods.** Study Population and Design, Protocols, Outcome Measures, and Statistical Analysis
eReferences.

Supplement 2.
Data Sharing Statement

## References

[1] Wang X, Liu J, Wang Q, . Economic-related inequalities in hepatitis B virus infection among 115.8 million pregnant women in China from 2013 to 2020. EClinicalMedicine. 2022;49:101465. doi:10.1016/j.eclinm.2022.101465 35747197 PMC9124701

[2] Kumar M, Abbas Z, Azami M, . Asian Pacific association for the study of liver (APASL) guidelines: hepatitis B virus in pregnancy. Hepatol Int. 2022;16(2):211-253. doi:10.1007/s12072-021-10285-5 35113359

[3] Mackens S, Santos-Ribeiro S, van de Vijver A, . Frozen embryo transfer: a review on the optimal endometrial preparation and timing. Hum Reprod. 2017;32(11):2234-2242. doi:10.1093/humrep/dex285 29025055

[4] Farsimadan M, Riahi SM, Muhammad HM, . The effects of hepatitis B virus infection on natural and IVF pregnancy: a meta-analysis study. J Viral Hepat. 2021;28(9):1234-1245. doi:10.1111/jvh.13565 34216533

[5] Wang L, Li L, Huang C, . Maternal chronic hepatitis B virus infection does not affect pregnancy outcomes in infertile patients receiving first in vitro fertilization treatment. Fertil Steril. 2019;112(2):250-257.e1. doi:10.1016/j.fertnstert.2019.03.039 31103286

[6] Auriti C, De Rose DU, Santisi A, . Pregnancy and viral infections: mechanisms of fetal damage, diagnosis and prevention of neonatal adverse outcomes from cytomegalovirus to SARS-CoV-2 and Zika virus. Biochim Biophys Acta Mol Basis Dis. 2021;1867(10):166198. doi:10.1016/j.bbadis.2021.166198 34118406 PMC8883330

