# Supplementary material for: Maternal Hepatitis B Virus Infection and Pregnancy Outcomes of Freeze-Thaw Embryo Transfer
Source: JAMA Netw Open. 2023 Jul 14;6(7):e2323495. doi: 10.1001/jamanetworkopen.2023.23495 (PMC10349339; doi:10.1001/jamanetworkopen.2023.23495)
Supplement: Supplement 1. — eMethods. Study Population and Design, Protocols, Outcome Measures, and Statistical Analysis eReferences. [file jamanetwopen-e2323495-s001.pdf]

# Supplementary Online Content

Ruan LL, Chen MX, Adu-Gyamfi EA, et al. Maternal hepatitis B virus infection and pregnancy outcomes of freeze-thaw embryo transfer. *JAMA Netw Open*. 2023;6(7):e2323495. doi:10.1001/jamanetworkopen.2023.23495

**eMethods**. Study Population and Design, Protocols, Outcome Measures, and Statistical Analysis
**eReferences**

This supplementary material has been provided by the authors to give readers additional information about their work.

© 2023 Ruan LL et al. *JAMA Network Open*.

**eMethods. Study Population and Design, Protocols, Outcome Measures, and Statistical Analysis**

**1.1. Study population and design**

This retrospective cohort study analyzed the data of women undergoing their first FET between January 2018 and April 2021. The electronic database at the Chengdu Xinan Gynecology Hospital and Chengdu Jinjiang Hospital for Women's and Children's Health was used for this work. The study excluded patients with abnormal karyotyping, hepatitis C, HIV, syphilis, history of recurrent abortion and implantation failure, missing lab data, and incomplete live birth information. Also, women who lacked HBV serostatus were excluded. The diagnostic criteria for HBV included the detection of HBsAg and HBeAg in the blood. HBsAg is the first marker of HBV infection. A positive HBsAg test result indicates that the person is infected with HBV. HBeAg, on the other hand, is a marker of high infectivity. A positive HBeAg test result suggests that the person is highly infectious and can transmit the virus to others. Based on the hepatitis B antigen exposure markers HBsAg and HBeAg, we divided the study population into three groups: (1) Group A (HBsAg+ HBeAg-) consisted of infected individuals who were not actively producing HBeAg. These people were in an inactive carrier state; (2) Group B (HBsAg+ HBeAg+) consisted of infected individuals actively producing both HBsAg and HBeAg. These people were in an active and highly infectious stage of hepatitis B infection; (3) Group C (HBsAg- HBeAg-): this group consisted of individuals who had not been infected with HBV or had been cured.

**1.2. Endometrial preparation protocols and embryo transfer strategy**

As detailed in previous studies [1-3], four endometrial preparation protocols were used. (1) Natural cycle (NC): the woman's menstrual cycle was monitored closely, and when the timing was right, a trigger medication was given to stimulate ovulation. (2) Hormone replacement cycle (HRC): on days 2–4 of the menstrual cycle, 4 mg estradiol valerate tablets (Progynova, Berlin, Germany) were given to the woman

© 2023 Ruan LL et al. *JAMA Network Open*.

to simulate the estrogen rise that occurs during a natural menstrual cycle. After ten days of estrogen treatment, progesterone was added to prepare the endometrium for implantation. (3) ovulation-promoting cycle (OPC): on days 2–4 of menstruation, 2.5–5 mg of letrozole or 20–40 mg of tamoxifen, or 50 mg of clomiphene were given to the woman to stimulate the ovaries to produce multiple follicles and promote ovulation. (4) Down-regulation cycle (DRC): on days 2-3 of the menstrual cycle, 3.75 mg of GnRH-a (Triptorelin, Ferring) was given to the woman. When the endometrium reached 8mm, the luteal transformation was initiated, and the highest quality cleavage-stage embryos or blastocysts were transferred 3-5 days later. Following embryo transfer, luteal support was provided.

The basic strategies for FET are summarized as follows: After the endometrium is prepared to receive the embryo, vitrified embryos are defrosted for implantation. It is recommended that only one or two embryos be transferred during FET to reduce the risk of multiple pregnancies. Based on the cryopreserved embryos of the patients, blastocyst transplantation was preferred, and cleavage-stage embryos were chosen exclusively in the absence of blastocysts. During the embryo transfer procedure, ultrasound guidance was used to ensure that the embryo was placed in the optimal location within the uterus. The support for the luteal phase commenced on the same day as oocyte retrieval 31. Luteal support was maintained until a negative pregnancy test was confirmed on the 14th day after the embryo transfer. In the case of successful conception, the administration of hormones was prolonged to 12 weeks of gestation.

**1.3. Outcome measures**

The primary outcome indicator of our study was live birth, and secondary outcomes were biochemical pregnancy, clinical pregnancy, ectopic pregnancy, miscarriage (including early miscarriage and late miscarriage), and singleton preterm delivery (< 37 gestational weeks). Live birth was defined as the

© 2023 Ruan LL et al. *JAMA Network Open*.

delivery of any living baby at or after 28 weeks of pregnancy. Biochemical pregnancy was defined as the serum β-HCG>25U/L after 14 days post embryo transfer. Clinical pregnancy was defined as the presence of a gestational sac or fetal heart confirmed by transvaginal ultrasound 28 days after embryo transfer. Ectopic pregnancy is when the fertilized egg is implanted and developed outside the uterus. Early miscarriage was described as a fetal loss within the first 12 gestational weeks, and late miscarriage was defined as a fetal loss within 12–28 gestational weeks.

**1.4. Statistical analysis**

To mitigate selection bias caused by confounding factors, we utilized propensity score matching (PSM) in this study. PSM enables the formation of balanced groups by matching individuals with similar characteristics. It is regarded as superior to traditional regression methods in reducing confounding bias. Through the matching process based on propensity scores, we ensured that the two groups had comparable baseline characteristics. The variables included in the PSM analysis were female age, BMI, duration of infertility, type of infertility, basal sex hormones (E2, P, FSH, LH), AMH, endometrial preparation protocols, endometrial thickness, the number of good quality embryos transferred, and the type of embryos transferred. A 1:4 nearest neighbor method was used to establish the matching ratios for Group A versus Group C, Group B versus Group C, and Group A versus Group B.

According to the type and distribution characteristics of the data, the baseline information and pregnancy outcomes in each group are presented as mean (SD), median (IQR) or number (N), and percentage (%). The differences between groups were compared using Student's t-tests, Mann-Whitney U, or Chi-square tests. Multivariate logistic regression was used to calculate each outcome's aORs and 95% confidence interval (CI). The regression analysis included multiple covariates. When comparing Groups A and C, we adjusted for propensity scores and LH levels, using Group C as the reference. In comparing Groups

© 2023 Ruan LL et al. *JAMA Network Open*.

B and C, we adjusted for propensity scores, using Group C as the reference group. In comparing Groups A and B, we adjusted for propensity scores, female age, and AMH levels, with Group A as the reference. We did perform a post hoc power calculation for the primary outcome (live birth). All statistical analyses were performed with SPSS version 25.0. P value <0.05 was considered to be statistically significant.

**eReferences.**


1. Wang Y, Hu WH, Wan Q, et al. Effect of artificial cycle with or without GnRH-a pretreatment on pregnancy and neonatal outcomes in women with PCOS after frozen embryo transfer: a propensity score matching study. *Reprod Biol Endocrinol.* 2022;20(1):56.
2. Hu YL, Wang Y, Geng LH, et al. Effects of sequential cleavage and blastocyst embryo transfer on pregnancy outcomes in patients with poor ovarian response. *J Reprod Immunol.* 2023;155:103780.
3. Ni ZX, Wan KM, Zhou ZH, Kuang YP, Yu CQ. Impact of Maternal Age on Singleton Birthweight in Frozen Embryo Transfer Cycles. *Front Endocrinol (Lausanne).* 2022;13:830414.


© 2023 Ruan LL et al. *JAMA Network Open*.
