# Supplementary material for: Maternal Hepatitis B Virus Infection and Pregnancy Outcomes of Freeze-Thaw Embryo Transfer
Source: JAMA Netw Open. 2023 Jul 14;6(7):e2323495. doi: 10.1001/jamanetworkopen.2023.23495 (PMC10349339; doi:10.1001/jamanetworkopen.2023.23495)
Supplement: Supplement 2. — Data Sharing Statement [file jamanetwopen-e2323495-s002.pdf]

Data Sharing Statement

Ruan. Maternal Hepatitis B Virus Infection and Pregnancy Outcomes of Freeze-Thaw Embryo Transfer. *JAMA Netw Open*. Published July 14, 2023.
doi:10.1001/jamanetworkopen.2023.23495

Data
Data available: No
